# Risks of severe agitation in pregnancy and its management

**DOI:** 10.1192/j.eurpsy.2026.10352

**Published:** 2026-07-31

**Authors:** A.-L. Sutter-Dallay

**Affiliations:** BPHRC INSERM 1219-HEALTHY Team, Charles Perrens Hospital and Bordeaux University, Bordeaux, France

## Abstract

**Abstract:**

While it is well known that the perinatal period is a time of risk for women’s mental health, the vast majority of episodes are mild to moderate in intensity. However, there are also episodes of severe decompensation that may be accompanied by auto- or hetero-aggressive behavioral disorders, which raises the question of appropriate care.The aim of this presentation is to describe the semiological contexts of these severe decompensations, as well as the therapeutic strategies involving medication and care pathways that can be proposed, with a focus on preventive care.

**Disclosure of Interest:**

None Declared

